# Supplementary material for: An Alliance of Polynitrogen Heterocycles: Novel Energetic Tetrazinedioxide-Hydroxytetrazole-Based Materials
Source: Molecules. 2022 Sep 11;27(18):5891. doi: 10.3390/molecules27185891 (PMC9505947; doi:10.3390/molecules27185891)
Supplement: Supplementary file 1 [file molecules-27-05891-s001.zip › molecules-1896044-supplementary.pdf]

Supporting Information for

An Alliance of Polynitrogen Heterocycles: Novel Energetic Tetrazinedioxide-Hydroxytetrazole-Based Materials

Dmitry M. Bystrov,[1] Alla N. Pivkina,[2] Leonid L. Fershtat[1*]

[1] N. D. Zelinsky Institute of Organic Chemistry, Russian Academy of Sciences
Leninsky Prosp. 47, Moscow 119991 (Russian Federation). E-mail: fershtat@ioc.ac.ru

[2] N. N. Semenov Federal Research Center for Chemical Physics, Russian Academy of Sciences, 4 Kosygin Str., Moscow 119991 (Russian Federation)

Table of Contents

S1. Crystallographic data 2

S2. Copies of NMR spectra 9

S1. Crystallographic data

Table S1. Crystal data and structure refinement for **11**.

Identification code	**11**	
Empirical formula	C3 H8 N10 O5	
Formula weight	264.19	
Temperature	100.0(1) K	
Wavelength	1.54184 Å	
Crystal system	Monoclinic	
Space group	$P\ 2_1/n$	
Unit cell dimensions	a = 9.22694(10) Å	α= 90°.
	b = 9.24085(7) Å	β= 107.6990(10)°.
	c = 11.66575(1) Å	γ = 90°.
Volume	947.596(14) $Å^3$	
Z	4	
Density (calculated)	1.852 g/cm^3	
Absorption coefficient	1.488 mm^{-1}	
F(000)	544	
Crystal size	0.22 x 0.11 x 0.09 mm^3	
Theta range for data collection	5.384 to 79.644°.	
Index ranges	-11<=h<=10, -11<=k<=7, -14<=l<=14	
Reflections collected	10971	
Independent reflections	2052 [R(int) = 0.0250]	
Observed reflections	1971	
Completeness to theta = 67.684°	99.9 %	
Absorption correction	Analytical	
Max. and min. transmission	0.900 and 0.808	
Refinement method	Full-matrix least-squares on F^2	
Data / restraints / parameters	2052 / 0 / 196	
Goodness-of-fit on F^2	1.060	
Final R indices [I>2sigma(I)]	R1 = 0.0290, wR2 = 0.0790	
R indices (all data)	R1 = 0.0299, wR2 = 0.0798	
Extinction coefficient	0.0018(3)	
Largest diff. peak and hole	0.366 and -0.231 e.$Å^{-3}$	

Table S2. Atomic coordinates (x 10^4) and equivalent isotropic displacement parameters (Å^{2}x 10^3) for **11**. U(eq) is defined as one third of the trace of the orthogonalized U^{ij} tensor.

	x	y	z	U(eq)
N(1)	2326(1)	5053(1)	5081(1)	11(1)
C(1)	3082(1)	4967(1)	4257(1)	11(1)
O(2)	6112(1)	6641(1)	2052(1)	15(1)
N(2)	1916(1)	3711(1)	5298(1)	14(1)
C(2)	3727(1)	6176(1)	3760(1)	11(1)
O(3)	3646(1)	9874(1)	3701(1)	15(1)
N(3)	2442(1)	2832(1)	4633(1)	14(1)
C(3)	4986(1)	8324(1)	2902(1)	11(1)
O(4)	1403(1)	9773(1)	4737(1)	17(1)
N(4)	3171(1)	3576(1)	3982(1)	13(1)
O(5)	-363(1)	7391(1)	2104(1)	15(1)
N(5)	4649(1)	5790(1)	3116(1)	12(1)
N(6)	5251(1)	6889(1)	2681(1)	11(1)
N(7)	4000(1)	8562(1)	3546(1)	11(1)
N(8)	3345(1)	7503(1)	3989(1)	12(1)
N(9)	5615(1)	9385(1)	2480(1)	14(1)
N(10)	510(1)	8500(1)	4425(1)	14(1)
O(1)	2041(1)	6178(1)	5671(1)	14(1)

Table S3. Bond lengths [Å] and angles [°] for **11**.

N(1)-C(1)	1.3507(14)
N(1)-N(2)	1.3426(13)
N(1)-O(1)	1.3171(12)
C(1)-C(2)	1.4653(14)
C(1)-N(4)	1.3331(14)
O(2)-N(6)	1.2559(12)
N(2)-N(3)	1.3136(14)
C(2)-N(5)	1.3429(13)
C(2)-N(8)	1.3256(14)
O(3)-N(7)	1.2828(12)
N(3)-N(4)	1.3470(13)
C(3)-N(6)	1.3869(14)
C(3)-N(7)	1.3620(13)
C(3)-N(9)	1.3092(14)
O(4)-N(10)	1.4185(12)
O(4)-H(4)	0.89(2)
O(5)-H(51)	0.85(2)
O(5)-H(52)	0.88(2)
N(5)-N(6)	1.3298(12)
N(7)-N(8)	1.3343(13)
N(9)-H(92)	0.886(19)
N(9)-H(91)	0.896(19)
N(10)-H(101)	0.933(17)
N(10)-H(102)	0.929(18)
N(10)-H(103)	0.926(18)
N(2)-N(1)-C(1)	108.44(9)
O(1)-N(1)-C(1)	130.27(9)
O(1)-N(1)-N(2)	121.21(9)
N(1)-C(1)-C(2)	126.62(9)
N(4)-C(1)-N(1)	108.08(9)
N(4)-C(1)-C(2)	125.29(10)
N(3)-N(2)-N(1)	106.51(9)
N(5)-C(2)-C(1)	114.91(9)
N(8)-C(2)-C(1)	117.44(9)

N(8)-C(2)-N(5)	127.65(10)
N(2)-N(3)-N(4)	110.74(9)
N(7)-C(3)-N(6)	116.22(9)
N(9)-C(3)-N(6)	121.51(10)
N(9)-C(3)-N(7)	122.23(10)
N(10)-O(4)-H(4)	107.4(13)
C(1)-N(4)-N(3)	106.21(9)
H(51)-O(5)-H(52)	104.1(18)
N(6)-N(5)-C(2)	114.82(9)
O(2)-N(6)-C(3)	117.49(9)
O(2)-N(6)-N(5)	119.71(9)
N(5)-N(6)-C(3)	122.80(9)
O(3)-N(7)-C(3)	118.17(9)
O(3)-N(7)-N(8)	118.27(8)
N(8)-N(7)-C(3)	123.52(9)
C(2)-N(8)-N(7)	114.90(9)
C(3)-N(9)-H(92)	118.3(11)
C(3)-N(9)-H(91)	118.4(12)
H(92)-N(9)-H(91)	123.2(17)
O(4)-N(10)-H(101)	112.7(10)
O(4)-N(10)-H(102)	110.5(10)
O(4)-N(10)-H(103)	105.1(11)
H(101)-N(10)-H(102)	108.7(14)
H(101)-N(10)-H(103)	109.8(15)
H(102)-N(10)-H(103)	110.0(15)

Table S4. Anisotropic displacement parameters ($Å^2$x 10^3) for **11**. The anisotropic displacement factor exponent takes the form: $-2\pi^2[h^2 a^{*2}U^{11} + ... + 2 h k a^* b^* U^{12}]$

	U^{11}	U^{22}	U^{33}	U^{23}	U^{13}	U^{12}
N(1)	12(1)	11(1)	12(1)	0(1)	5(1)	0(1)
C(1)	11(1)	12(1)	12(1)	1(1)	5(1)	1(1)
O(2)	17(1)	16(1)	16(1)	0(1)	12(1)	2(1)
N(2)	15(1)	12(1)	15(1)	1(1)	5(1)	-2(1)
C(2)	11(1)	12(1)	10(1)	0(1)	3(1)	0(1)
O(3)	19(1)	8(1)	21(1)	0(1)	12(1)	2(1)
N(3)	16(1)	12(1)	15(1)	1(1)	6(1)	-2(1)
C(3)	11(1)	12(1)	11(1)	1(1)	5(1)	1(1)
O(4)	19(1)	12(1)	26(1)	-4(1)	14(1)	-3(1)
N(4)	15(1)	11(1)	14(1)	1(1)	5(1)	-1(1)
O(5)	16(1)	14(1)	17(1)	2(1)	8(1)	1(1)
N(5)	14(1)	11(1)	13(1)	2(1)	6(1)	0(1)
N(6)	12(1)	12(1)	11(1)	1(1)	6(1)	1(1)
N(7)	12(1)	10(1)	13(1)	1(1)	6(1)	0(1)
N(8)	13(1)	11(1)	13(1)	2(1)	6(1)	0(1)
N(9)	17(1)	12(1)	17(1)	0(1)	10(1)	0(1)
N(10)	15(1)	12(1)	17(1)	-1(1)	7(1)	0(1)
O(1)	17(1)	12(1)	15(1)	-2(1)	10(1)	1(1)

Table S5. Hydrogen coordinates (x 10^4) and isotropic displacement parameters ($Å^2$x 10^3) for **11**.

	x	y	z	U(eq)
H(92)	5482(19)	10280(20)	2699(15)	26(4)
H(101)	197(18)	8322(18)	3599(16)	23(4)
H(91)	6190(20)	9170(20)	2012(17)	35(5)
H(102)	1049(18)	7701(19)	4818(15)	24(4)
H(103)	-330(20)	8662(19)	4684(16)	29(4)
H(4)	2120(20)	9730(20)	4368(18)	36(5)
H(51)	310(20)	7630(20)	1776(17)	34(5)
H(52)	-1200(20)	7830(20)	1664(19)	43(5)

Table S6. Torsion angles [°] for **11**.

N(1)-C(1)-C(2)-N(5)	168.90(10)
N(1)-C(1)-C(2)-N(8)	-11.07(16)
N(1)-C(1)-N(4)-N(3)	0.95(11)
N(1)-N(2)-N(3)-N(4)	-0.59(11)
C(1)-N(1)-N(2)-N(3)	1.17(11)
C(1)-C(2)-N(5)-N(6)	-179.03(8)
C(1)-C(2)-N(8)-N(7)	178.09(9)
N(2)-N(1)-C(1)-C(2)	179.79(9)
N(2)-N(1)-C(1)-N(4)	-1.33(12)
N(2)-N(3)-N(4)-C(1)	-0.23(11)
C(2)-C(1)-N(4)-N(3)	179.85(9)
C(2)-N(5)-N(6)-O(2)	-178.97(9)
C(2)-N(5)-N(6)-C(3)	1.84(14)
O(3)-N(7)-N(8)-C(2)	177.85(9)
C(3)-N(7)-N(8)-C(2)	0.08(14)
N(4)-C(1)-C(2)-N(5)	-9.80(15)
N(4)-C(1)-C(2)-N(8)	170.23(10)
N(5)-C(2)-N(8)-N(7)	-1.88(15)
N(6)-C(3)-N(7)-O(3)	-175.44(9)
N(6)-C(3)-N(7)-N(8)	2.34(15)
N(7)-C(3)-N(6)-O(2)	177.43(8)
N(7)-C(3)-N(6)-N(5)	-3.36(15)
N(8)-C(2)-N(5)-N(6)	0.94(16)
N(9)-C(3)-N(6)-O(2)	-0.41(15)
N(9)-C(3)-N(6)-N(5)	178.80(9)
N(9)-C(3)-N(7)-O(3)	2.38(15)
N(9)-C(3)-N(7)-N(8)	-179.84(10)
O(1)-N(1)-C(1)-C(2)	-3.49(18)
O(1)-N(1)-C(1)-N(4)	175.39(10)
O(1)-N(1)-N(2)-N(3)	-175.91(9)

Table S7. Hydrogen bonds for **11** [Å and °].

D-H...A	d(D-H)	d(H...A)	d(D...A)	<(DHA)
N(9)-H(92)...O(5)#1	0.886(19)	1.971(19)	2.8420(13)	167.4(16)
N(10)-H(101)...O(5)	0.933(17)	1.870(17)	2.7752(13)	162.8(15)
N(9)-H(91)...O(1)#2	0.896(19)	1.98(2)	2.8538(12)	166.5(17)
N(10)-H(102)...O(1)	0.929(18)	1.804(18)	2.7321(12)	175.8(16)
N(10)-H(103)...O(4)#3	0.926(18)	1.983(18)	2.7692(13)	141.5(15)
O(4)-H(4)...O(3)	0.89(2)	1.81(2)	2.6965(11)	172.8(19)
O(4)-H(4)...N(8)	0.89(2)	2.45(2)	3.0534(12)	124.9(16)
O(5)-H(51)...N(4)#1	0.85(2)	2.07(2)	2.9074(13)	169.7(18)
O(5)-H(52)...O(1)#4	0.88(2)	1.92(2)	2.8011(11)	178(2)

Symmetry transformations used to generate equivalent atoms:

#1 -x+1/2,y+1/2,-z+1/2 #2 x+1/2,-y+3/2,z-1/2

#3 -x,-y+2,-z+1 #4 x-1/2,-y+3/2,z-1/2

S2. Copies of NMR spectra

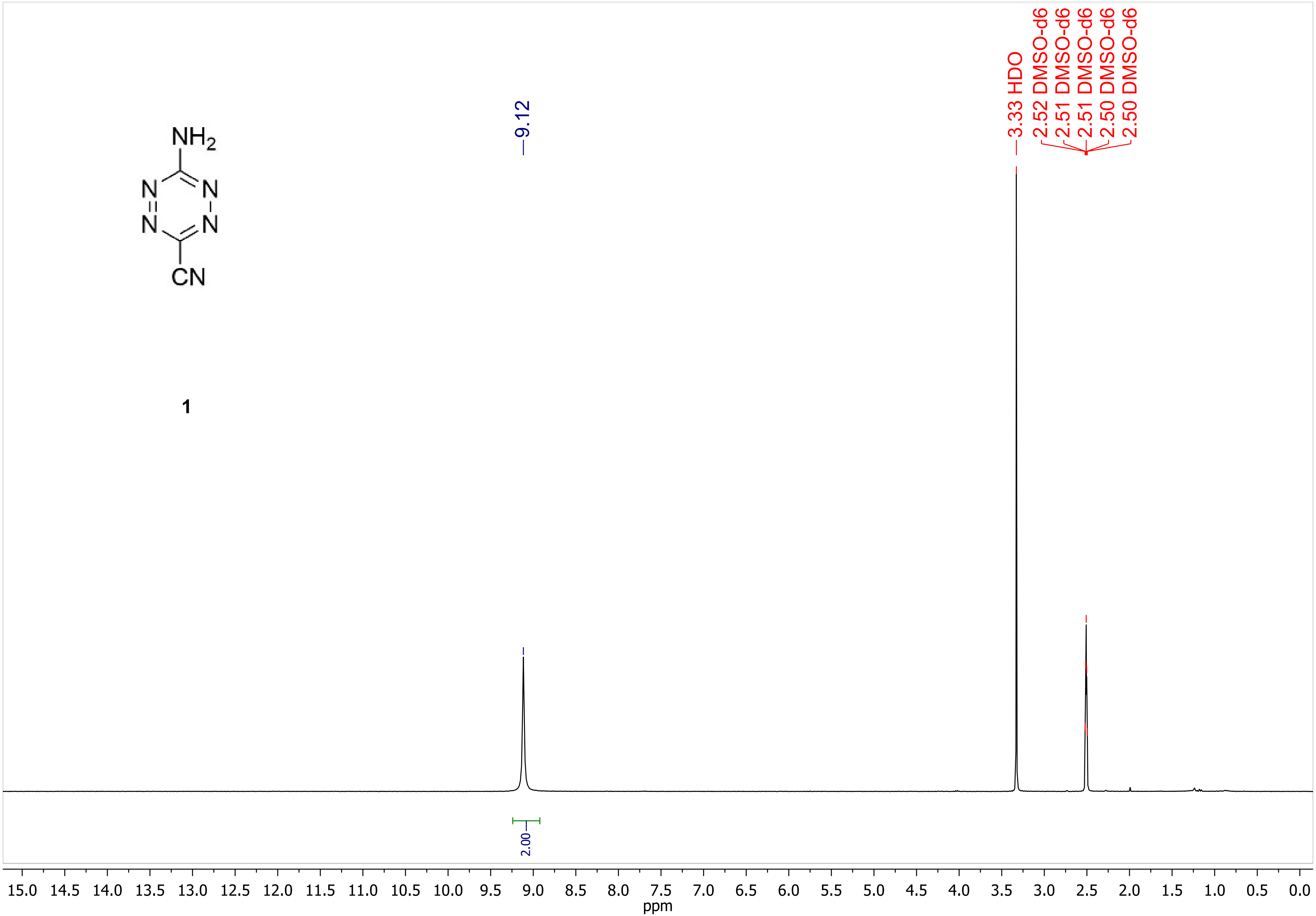

NH2
N
N
N
N
CN
1
9.12
3.33 HDO
2.52 DMSO-d6
2.51 DMSO-d6
2.51 DMSO-d6
2.50 DMSO-d6
2.50 DMSO-d6
2.00
15.0 14.5 14.0 13.5 13.0 12.5 12.0 11.5 11.0 10.5 10.0 9.5 9.0 8.5 8.0 7.5 7.0 6.5 6.0 5.5 5.0 4.5 4.0 3.5 3.0 2.5 2.0 1.5 1.0 0.5 0.0
ppm

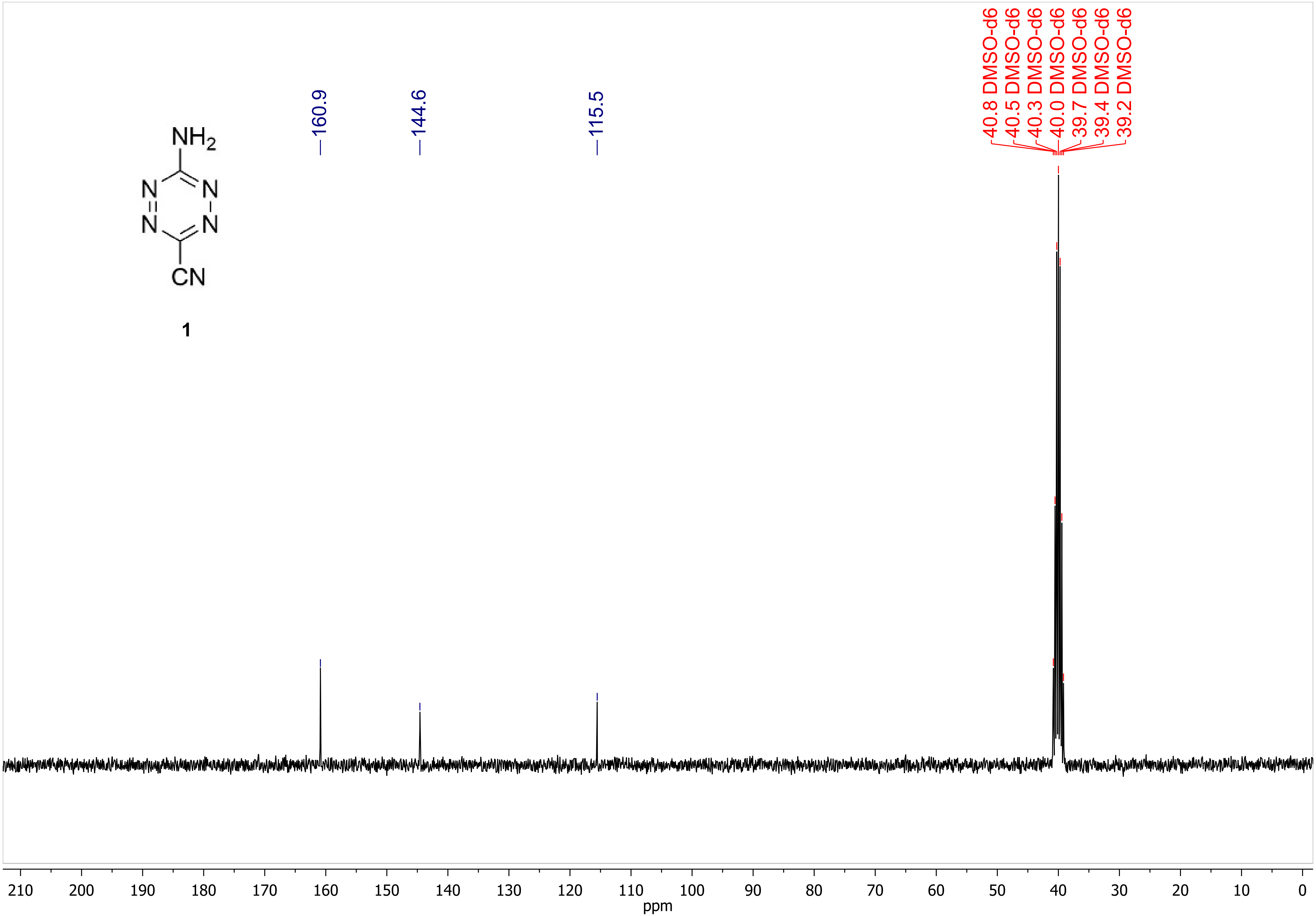

NH2
N
N
N
N
CN
1
160.9
144.6
115.5
40.8 DMSO-d6
40.5 DMSO-d6
40.3 DMSO-d6
40.0 DMSO-d6
39.7 DMSO-d6
39.4 DMSO-d6
39.2 DMSO-d6
210 200 190 180 170 160 150 140 130 120 110 100 90 80 70 60 50 40 30 20 10 0
ppm

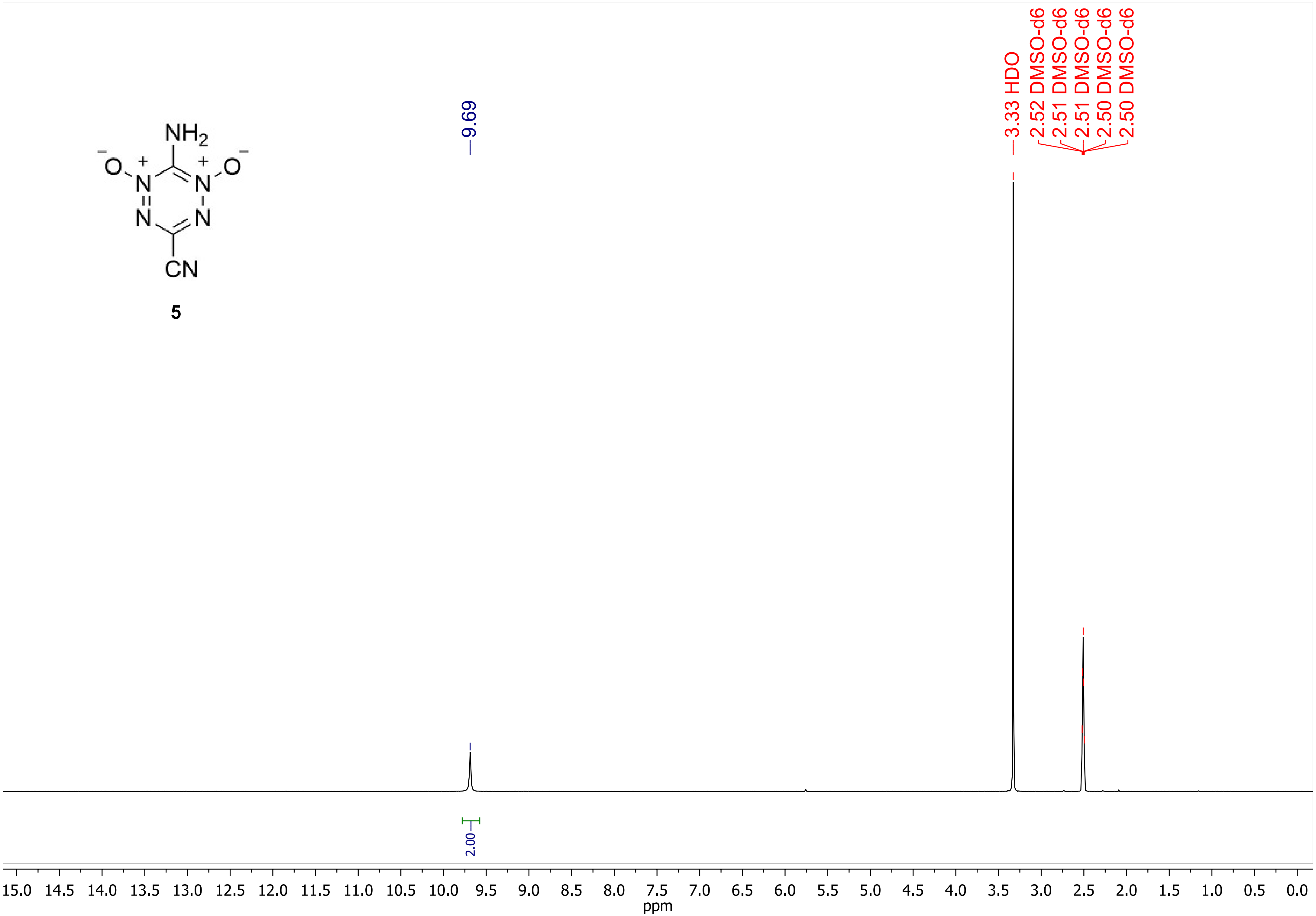
NH2
O
N
N
N
N
O
CN
5
9.69
3.33 HDO
2.52 DMSO-d6
2.51 DMSO-d6
2.51 DMSO-d6
2.50 DMSO-d6
2.50 DMSO-d6
2.00
15.0 14.5 14.0 13.5 13.0 12.5 12.0 11.5 11.0 10.5 10.0 9.5 9.0 8.5 8.0 7.5 7.0 6.5 6.0 5.5 5.0 4.5 4.0 3.5 3.0 2.5 2.0 1.5 1.0 0.5 0.0
ppm

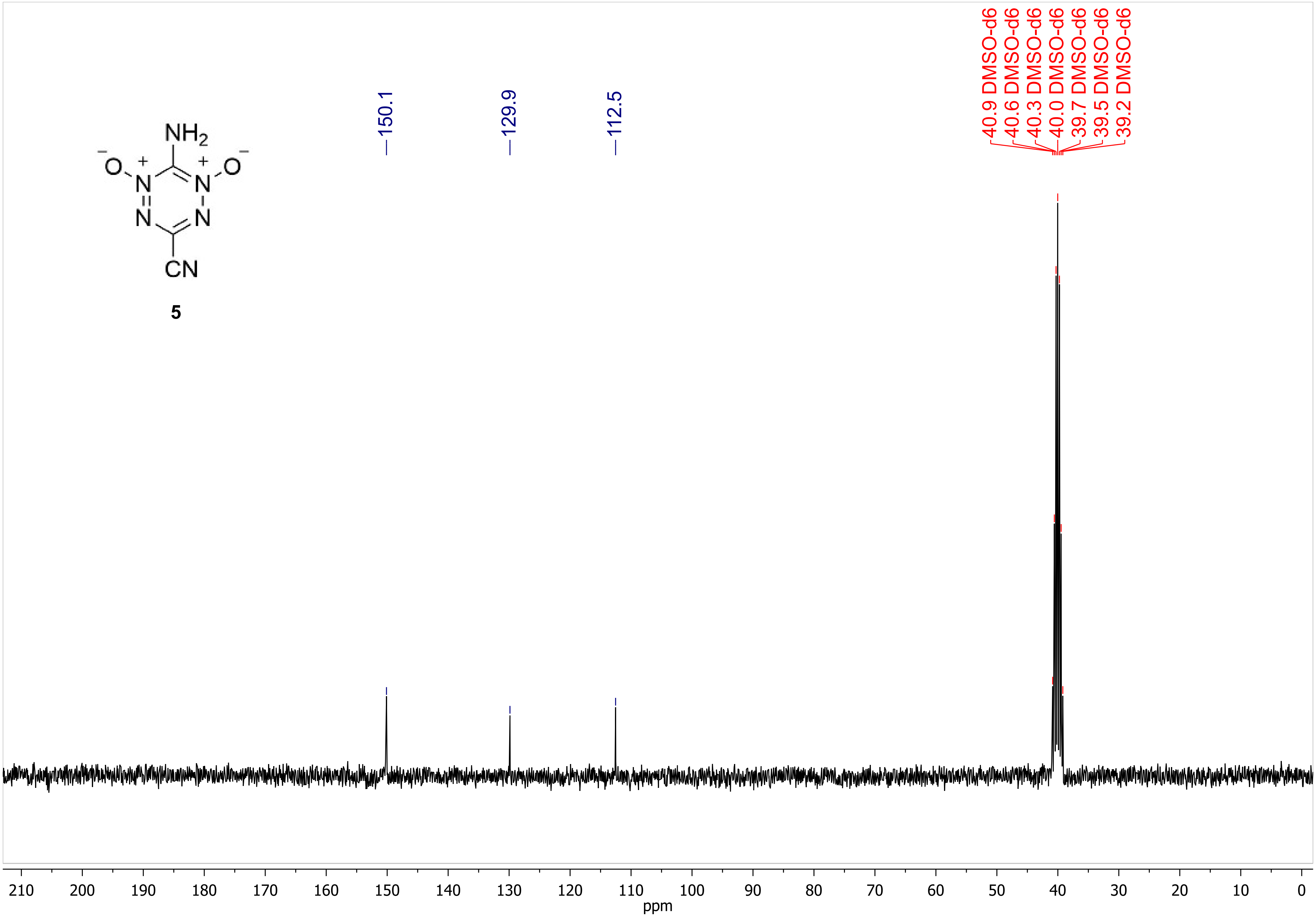

150.1
129.9
112.5
40.9 DMSO-d6
40.6 DMSO-d6
40.3 DMSO-d6
40.0 DMSO-d6
39.7 DMSO-d6
39.5 DMSO-d6
39.2 DMSO-d6
NH2
CN
ppm

5

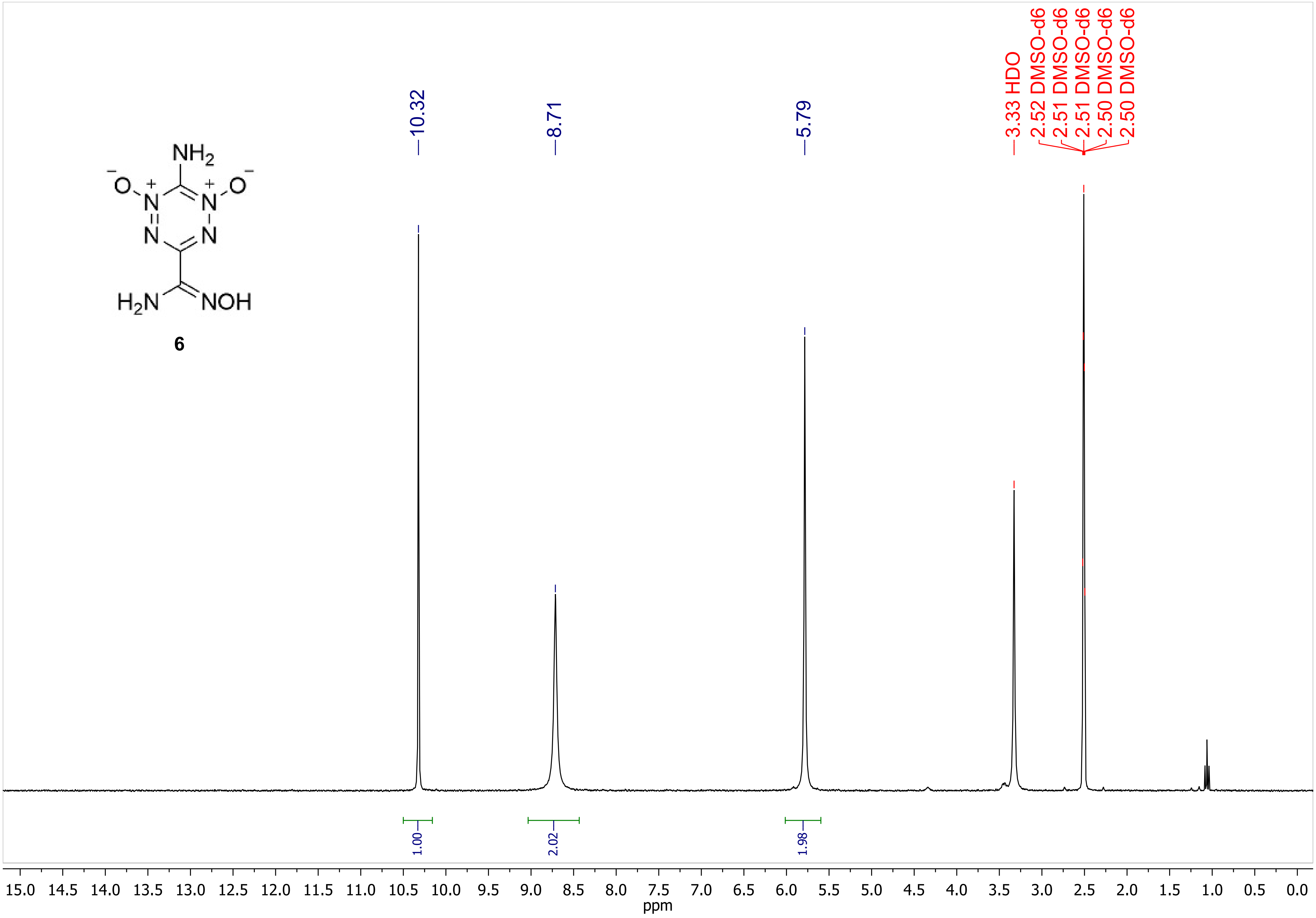

10.32
8.71
5.79
3.33 HDO
2.52 DMSO-d6
2.51 DMSO-d6
2.51 DMSO-d6
2.50 DMSO-d6
2.50 DMSO-d6
NH2
O
N
N
N
N
H2N
NOH
6
1.00
2.02
1.98
15.0 14.5 14.0 13.5 13.0 12.5 12.0 11.5 11.0 10.5 10.0 9.5 9.0 8.5 8.0 7.5 7.0 6.5 6.0 5.5 5.0 4.5 4.0 3.5 3.0 2.5 2.0 1.5 1.0 0.5 0.0
ppm

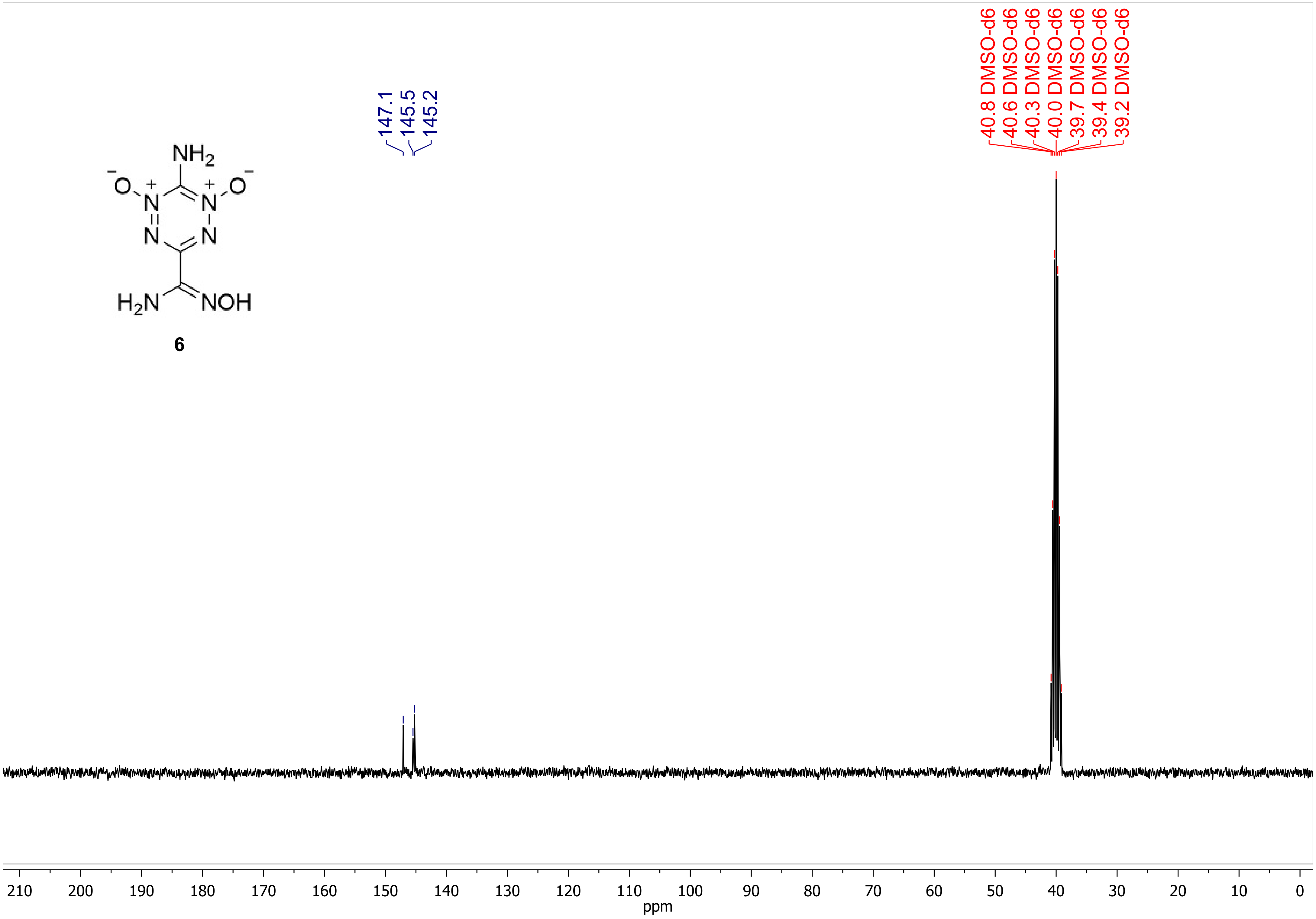

147.1
145.5
145.2
40.8 DMSO-d6
40.6 DMSO-d6
40.3 DMSO-d6
40.0 DMSO-d6
39.7 DMSO-d6
39.4 DMSO-d6
39.2 DMSO-d6
NH2
O
N
N
N
N
H2N
NOH
6
210
200
190
180
170
160
150
140
130
120
110
100
90
80
70
60
50
40
30
20
10
0
ppm

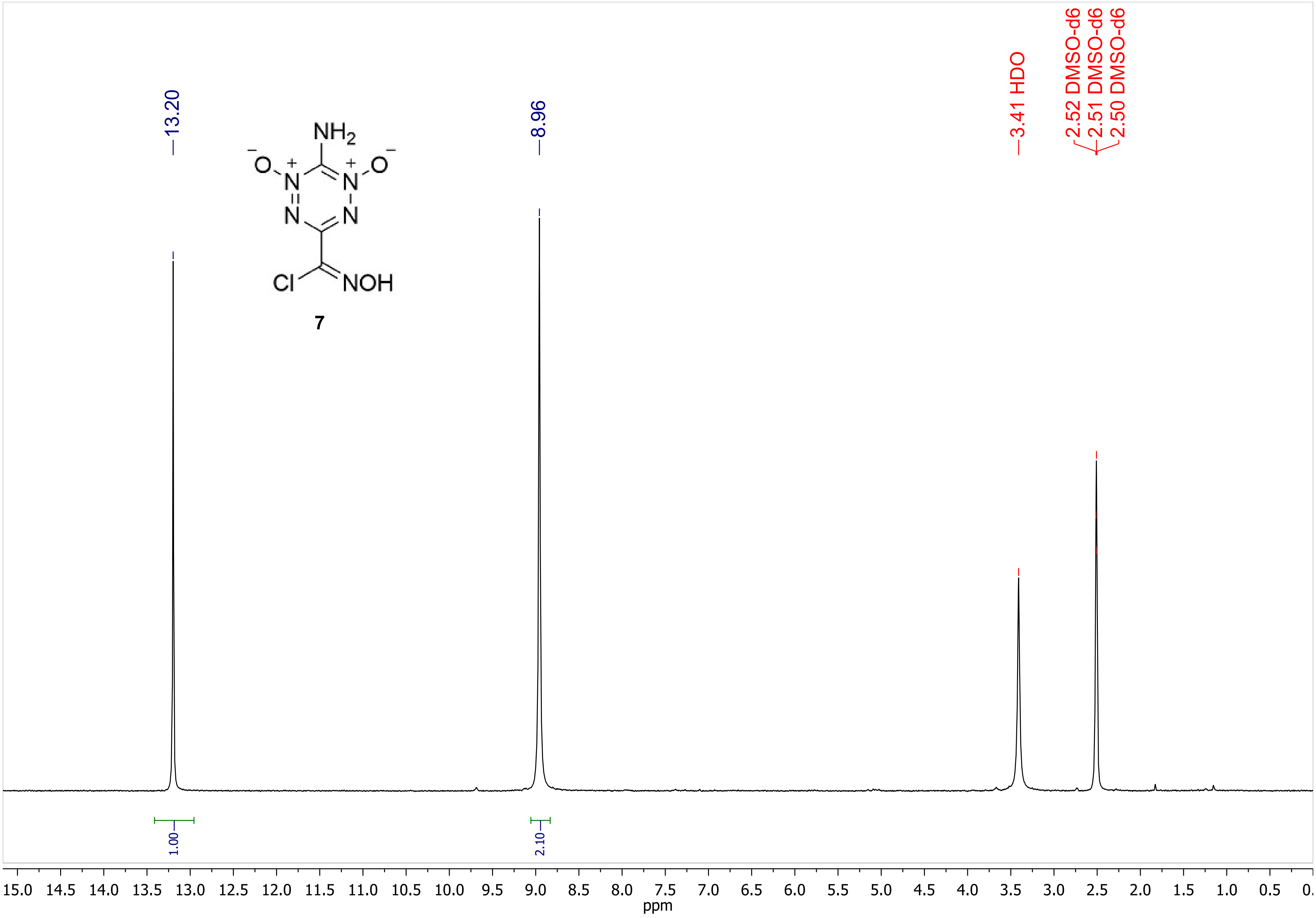

13.20
8.96
3.41 HDO
2.52 DMSO-d6
2.51 DMSO-d6
2.50 DMSO-d6
NH2
O
N
N
N
N
O
Cl
NOH
7
1.00
2.10
15.0 14.5 14.0 13.5 13.0 12.5 12.0 11.5 11.0 10.5 10.0 9.5 9.0 8.5 8.0 7.5 7.0 6.5 6.0 5.5 5.0 4.5 4.0 3.5 3.0 2.5 2.0 1.5 1.0 0.5 0.
ppm

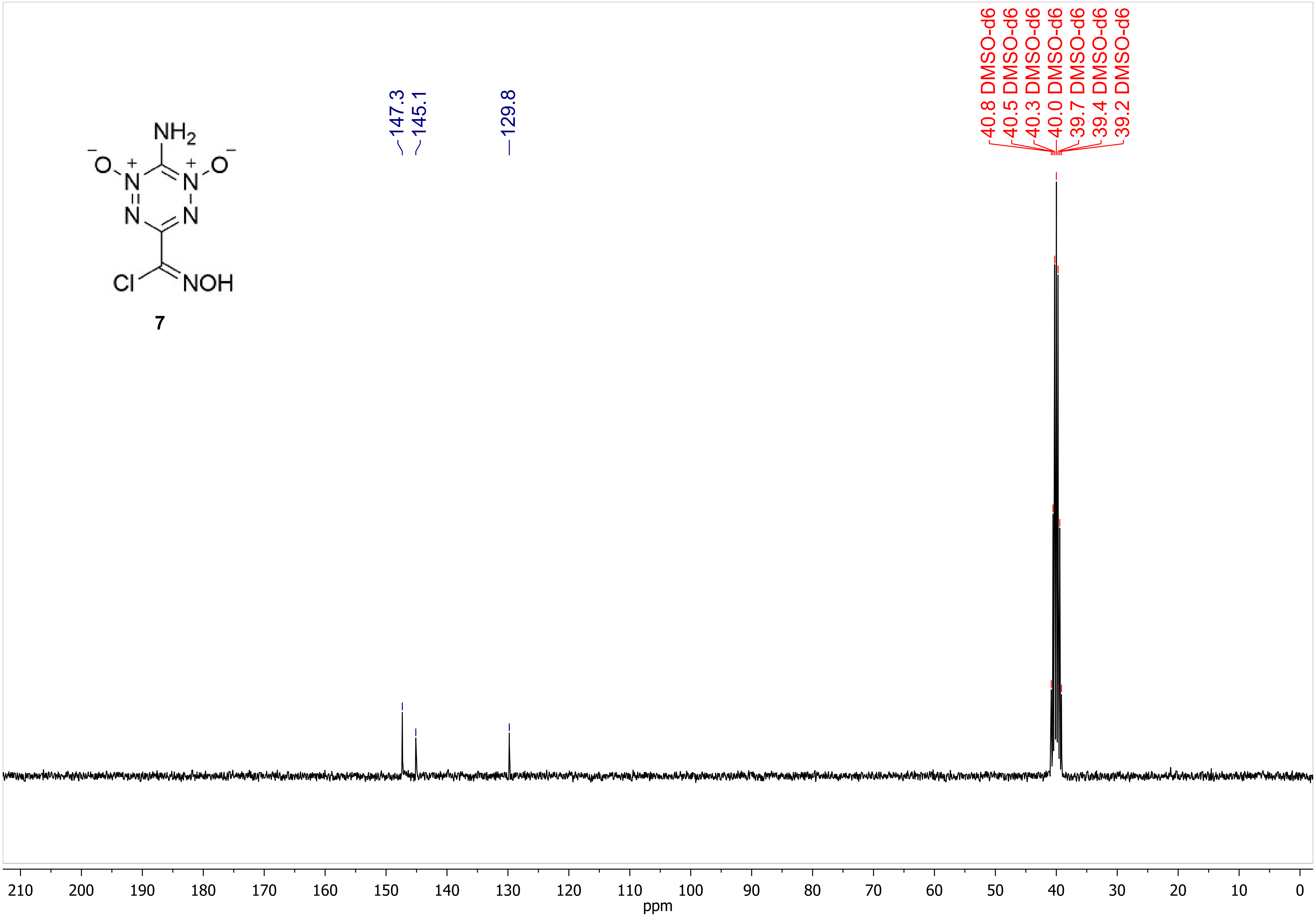

147.3
145.1
129.8
40.8 DMSO-d6
40.5 DMSO-d6
40.3 DMSO-d6
40.0 DMSO-d6
39.7 DMSO-d6
39.4 DMSO-d6
39.2 DMSO-d6
NH2
O
N
N
N
N
O
Cl
NOH
7
210
200
190
180
170
160
150
140
130
120
110
100
90
80
70
60
50
40
30
20
10
0
ppm

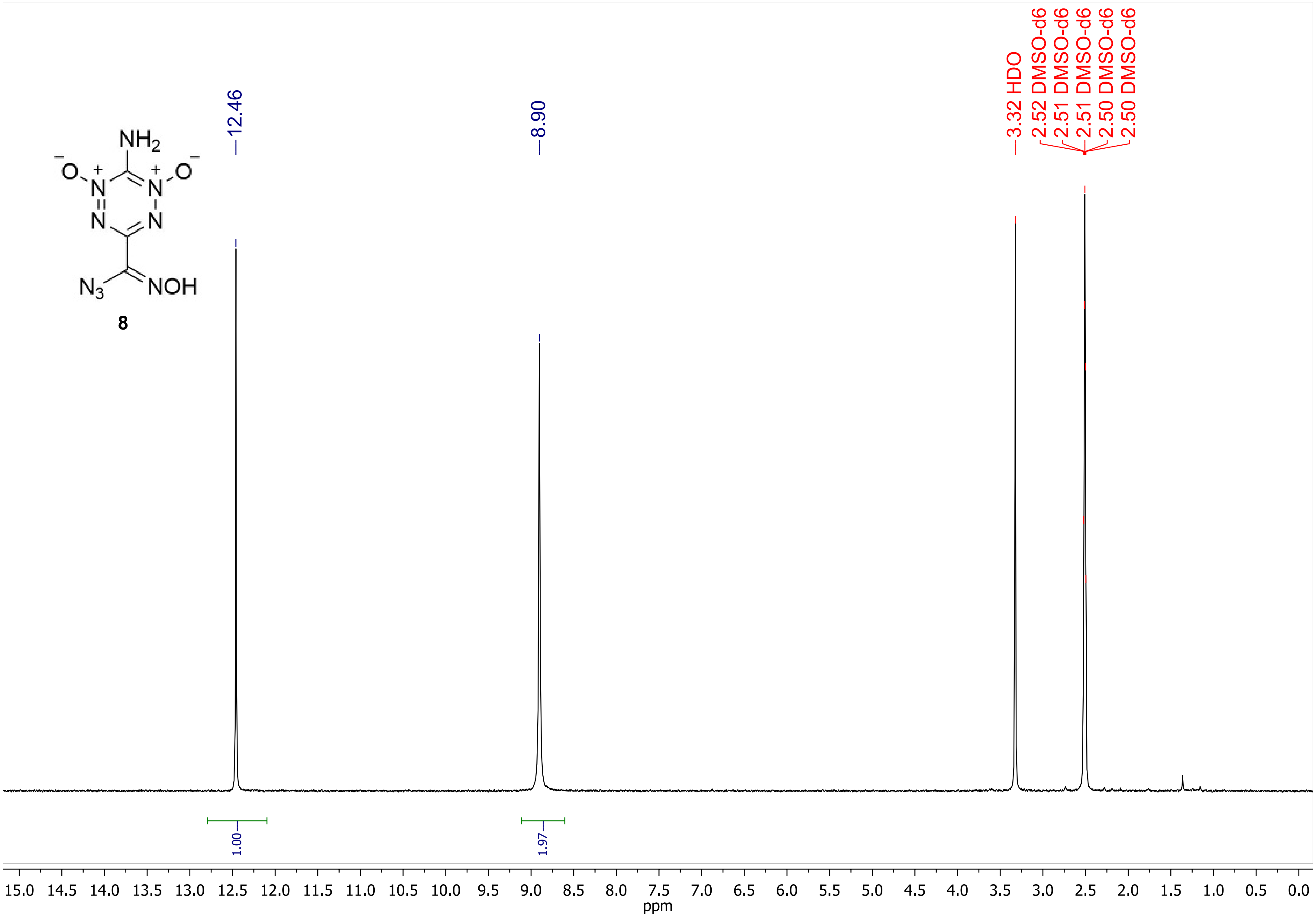

NH2
O
N
N
N
N
N3
NOH
8
12.46
8.90
3.32 HDO
2.52 DMSO-d6
2.51 DMSO-d6
2.51 DMSO-d6
2.50 DMSO-d6
2.50 DMSO-d6
1.00
1.97
15.0 14.5 14.0 13.5 13.0 12.5 12.0 11.5 11.0 10.5 10.0 9.5 9.0 8.5 8.0 7.5 7.0 6.5 6.0 5.5 5.0 4.5 4.0 3.5 3.0 2.5 2.0 1.5 1.0 0.5 0.0
ppm

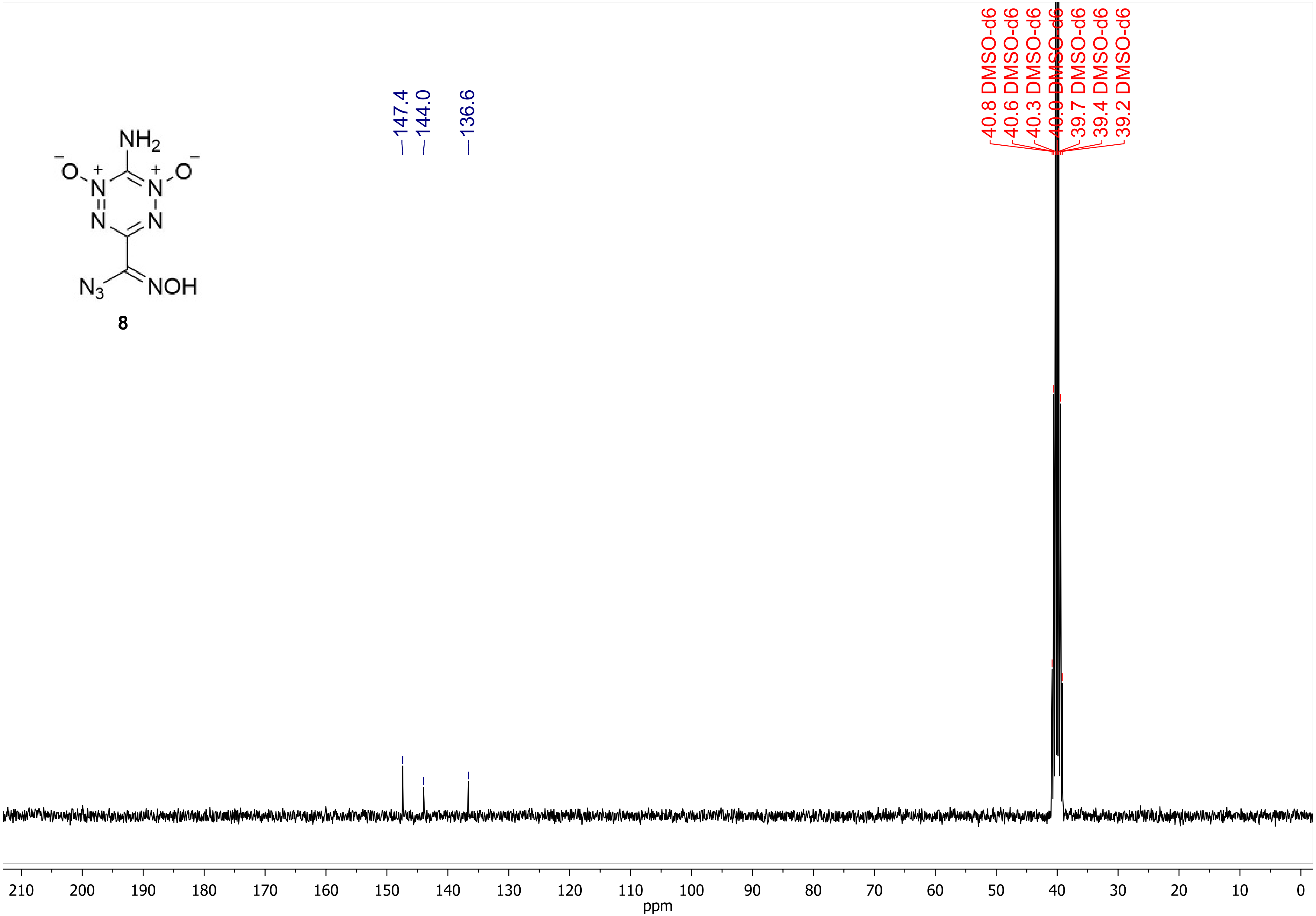

NH2
N3
NOH
8
147.4
144.0
136.6
40.8 DMSO-d6
40.6 DMSO-d6
40.3 DMSO-d6
40.0 DMSO-d6
39.7 DMSO-d6
39.4 DMSO-d6
39.2 DMSO-d6
210 200 190 180 170 160 150 140 130 120 110 100 90 80 70 60 50 40 30 20 10 0
ppm

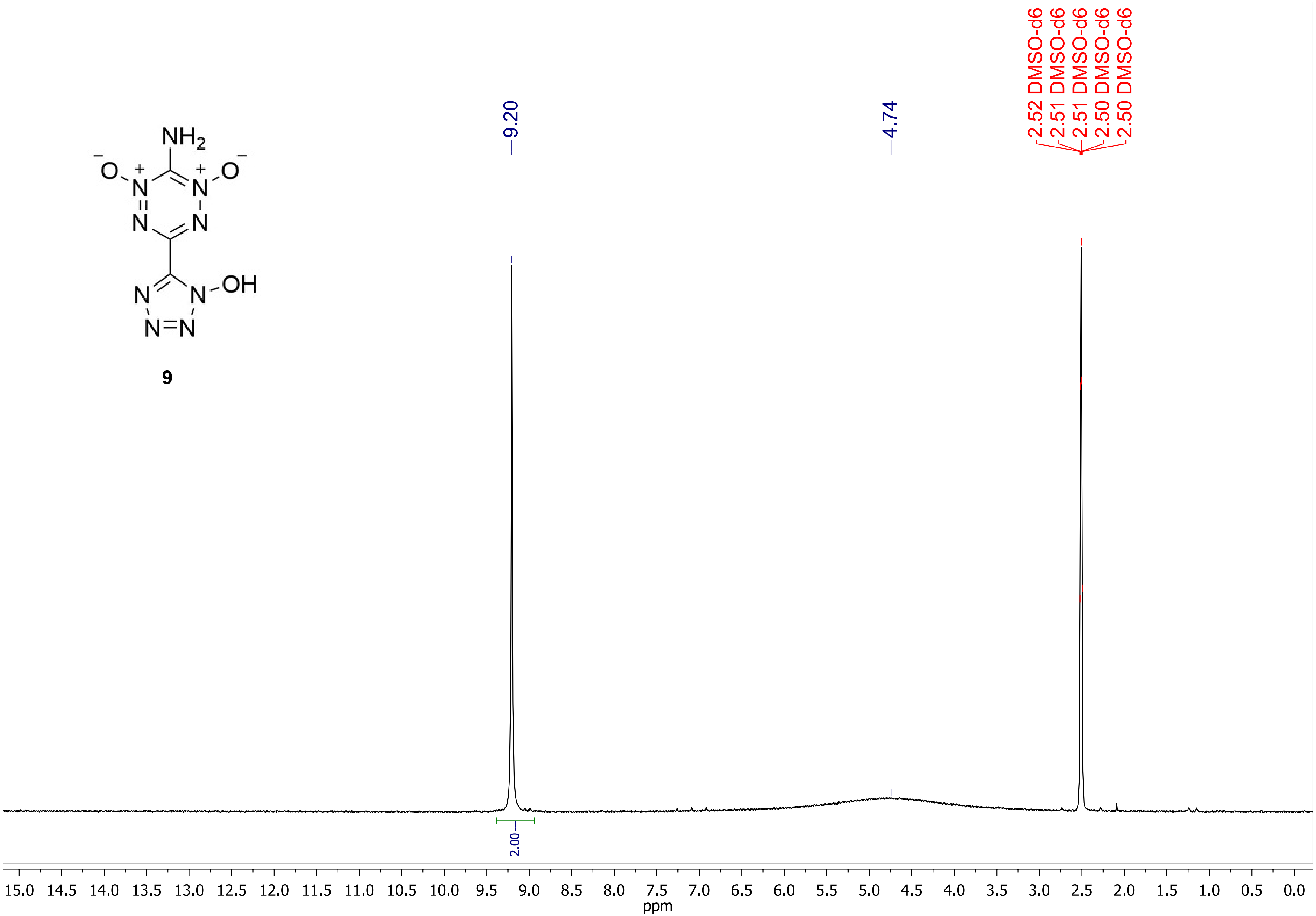
9.20
4.74
2.52 DMSO-d6
2.51 DMSO-d6
2.51 DMSO-d6
2.50 DMSO-d6
2.50 DMSO-d6
2.00
15.0 14.5 14.0 13.5 13.0 12.5 12.0 11.5 11.0 10.5 10.0 9.5 9.0 8.5 8.0 7.5 7.0 6.5 6.0 5.5 5.0 4.5 4.0 3.5 3.0 2.5 2.0 1.5 1.0 0.5 0.0
ppm
NH2
O
N
N
N
N
N
N
N
N
OH
9

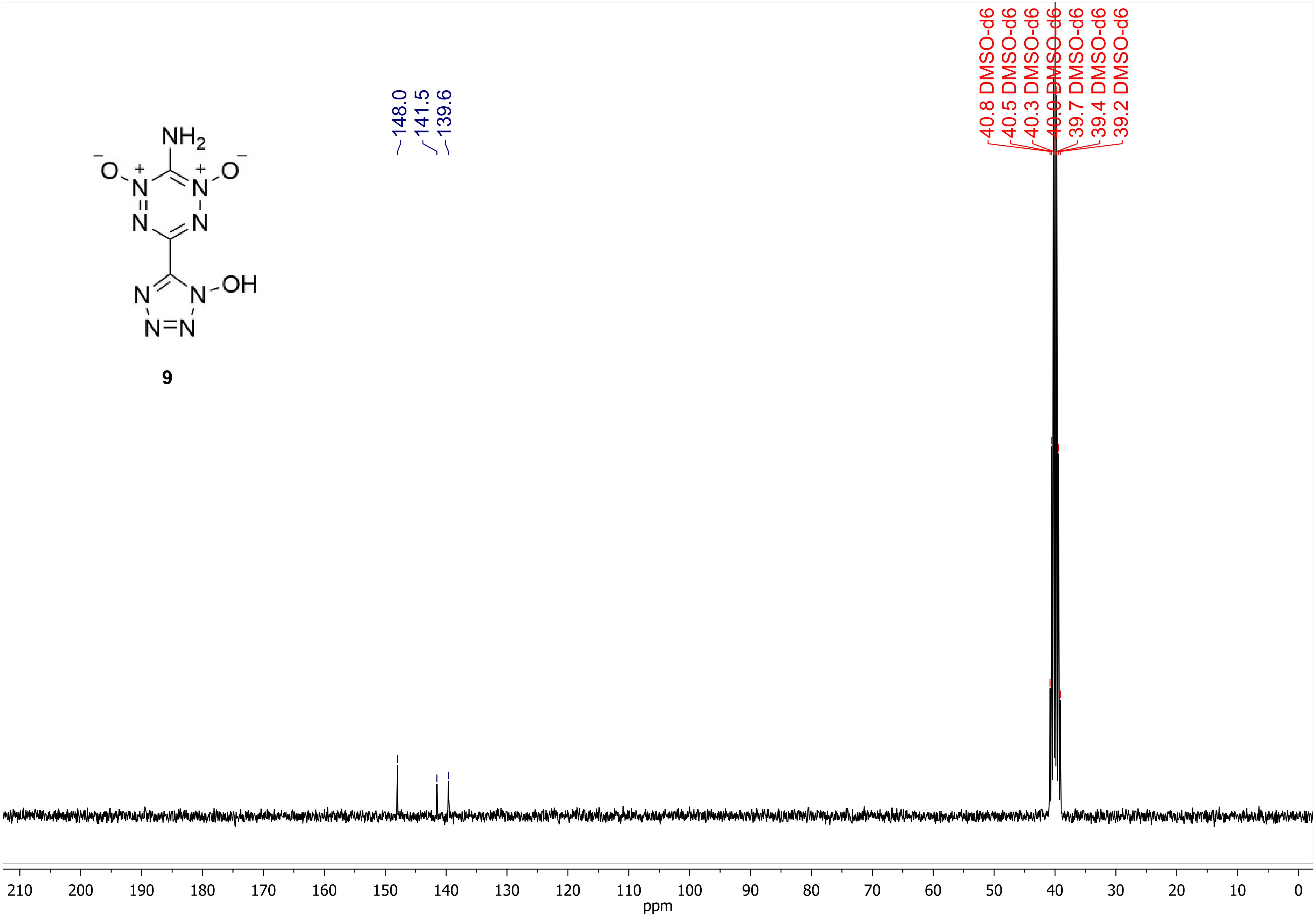

NH2
O
N
N
N
N
O
N
OH
N
N=N
148.0
141.5
139.6
40.8 DMSO-d6
40.5 DMSO-d6
40.3 DMSO-d6
40.0 DMSO-d6
39.7 DMSO-d6
39.4 DMSO-d6
39.2 DMSO-d6
210
200
190
180
170
160
150
140
130
120
110
100
90
80
70
60
50
40
30
20
10
0
ppm

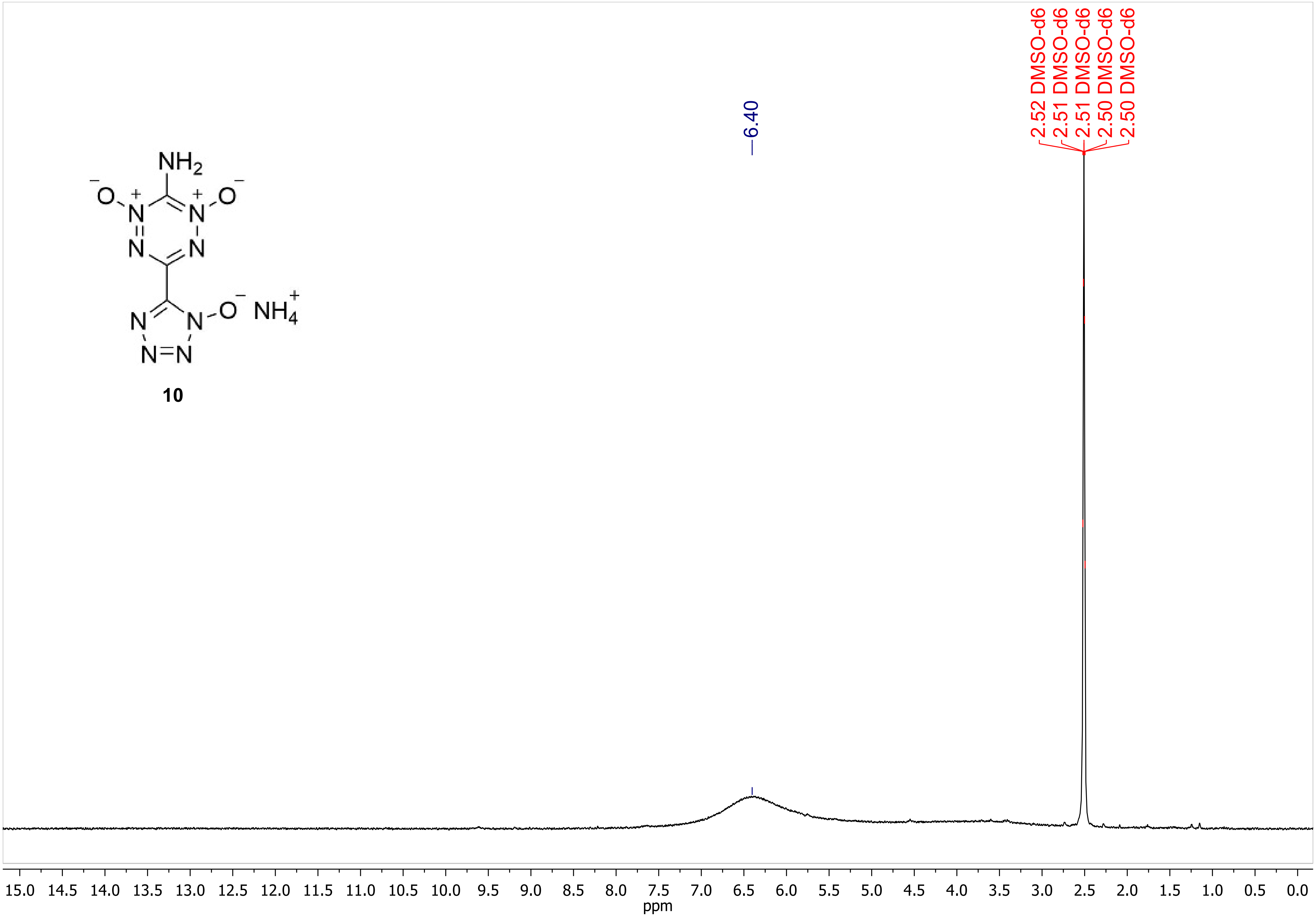

NH2
O N N O
N N
N N O NH4
N=N
10
—6.40
2.52 DMSO-d6
2.51 DMSO-d6
2.51 DMSO-d6
2.50 DMSO-d6
2.50 DMSO-d6
15.0 14.5 14.0 13.5 13.0 12.5 12.0 11.5 11.0 10.5 10.0 9.5 9.0 8.5 8.0 7.5 7.0 6.5 6.0 5.5 5.0 4.5 4.0 3.5 3.0 2.5 2.0 1.5 1.0 0.5 0.0
ppm

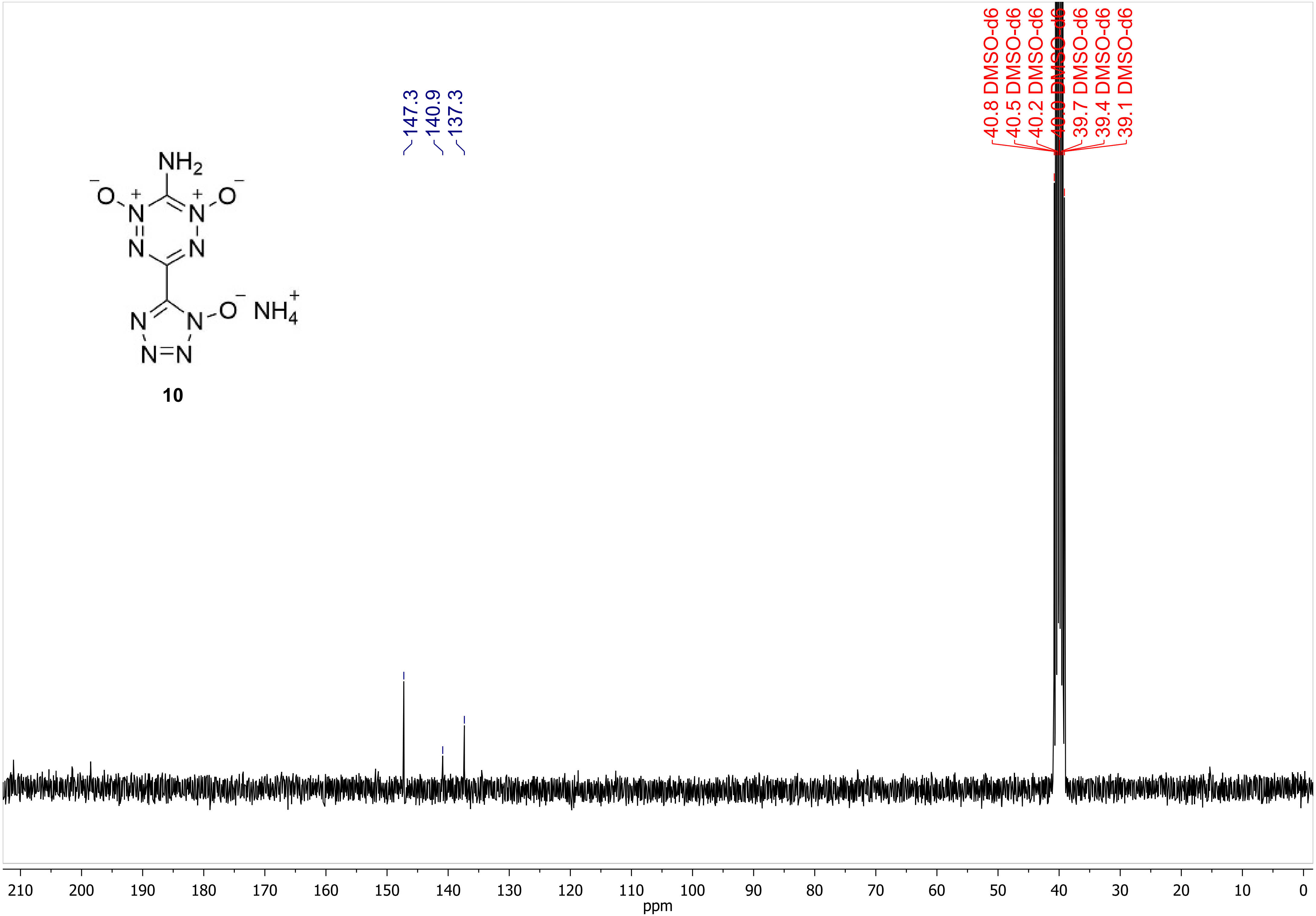
147.3
140.9
137.3
40.8 DMSO-d6
40.5 DMSO-d6
40.2 DMSO-d6
40.0 DMSO-d6
39.7 DMSO-d6
39.4 DMSO-d6
39.1 DMSO-d6
NH_2
O
N
N
N
N
O
N
N
N
N
O
NH_4^+
10
210
200
190
180
170
160
150
140
130
120
110
100
90
80
70
60
50
40
30
20
10
0
ppm

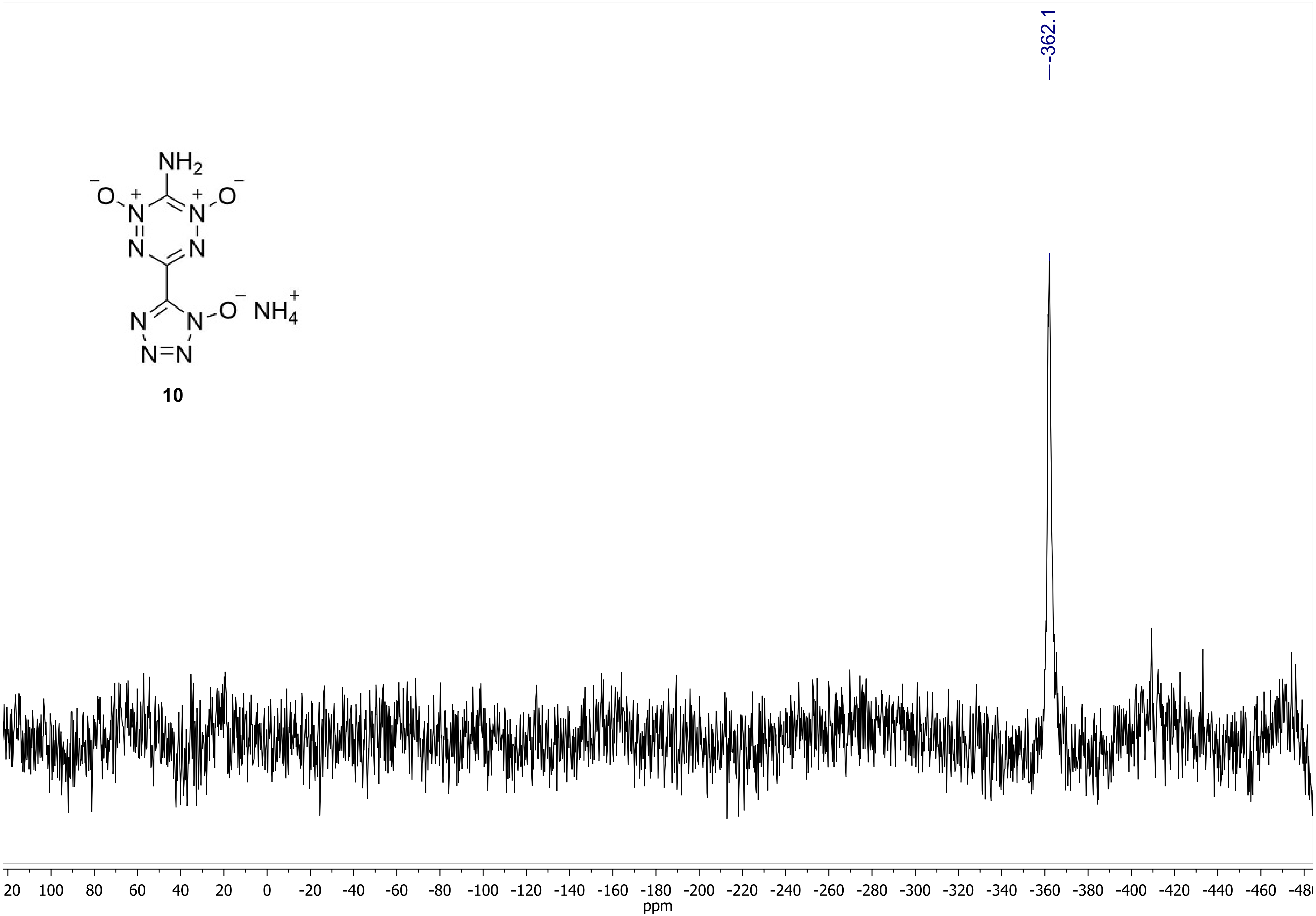
-362.1
NH2
O N N O
N N
N N O NH4
N N N
10
20 100 80 60 40 20 0 -20 -40 -60 -80 -100 -120 -140 -160 -180 -200 -220 -240 -260 -280 -300 -320 -340 -360 -380 -400 -420 -440 -460 -48
ppm

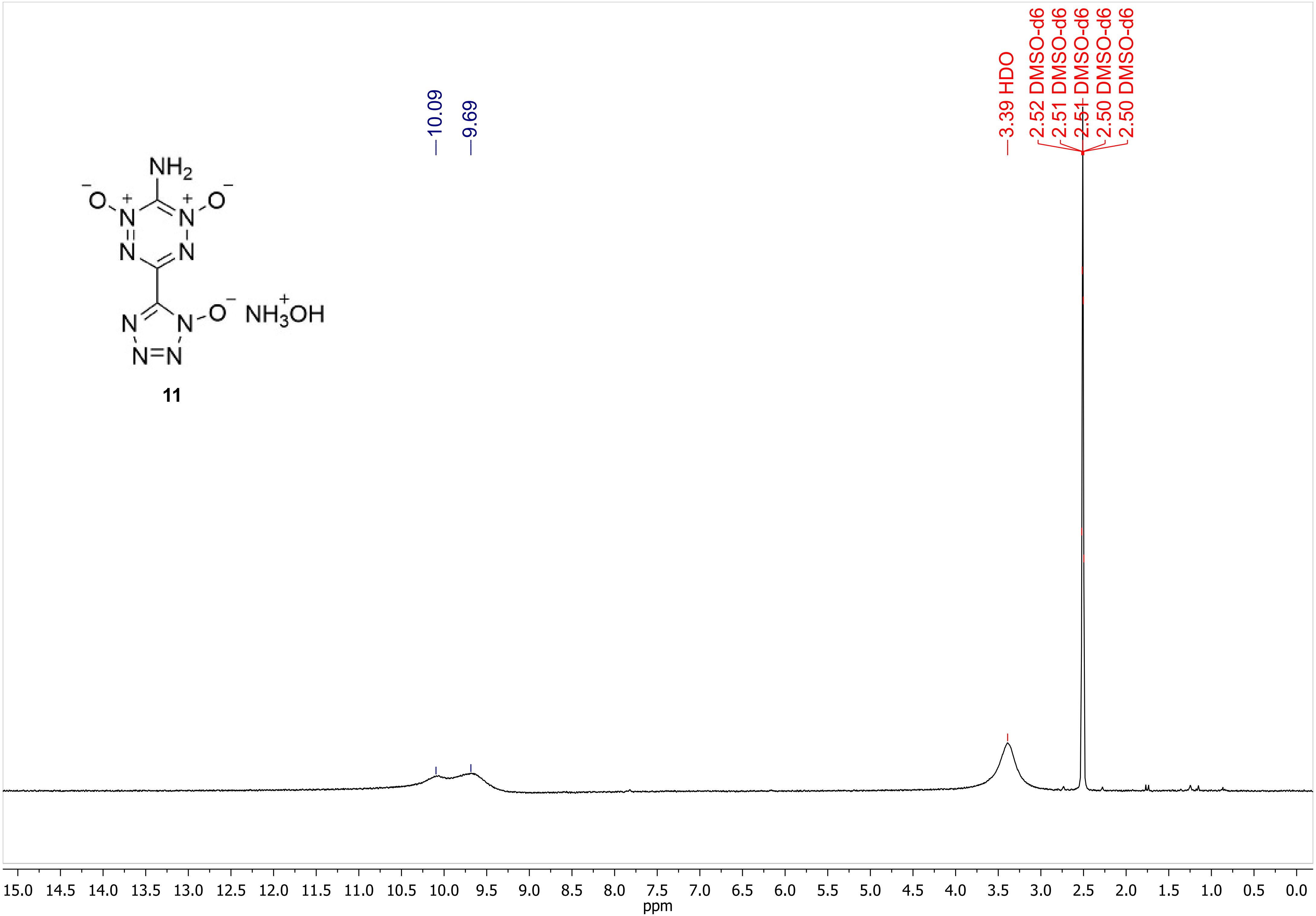
10.09
9.69
3.39 HDO
2.52 DMSO-d6
2.51 DMSO-d6
2.51 DMSO-d6
2.50 DMSO-d6
2.50 DMSO-d6
15.0 14.5 14.0 13.5 13.0 12.5 12.0 11.5 11.0 10.5 10.0 9.5 9.0 8.5 8.0 7.5 7.0 6.5 6.0 5.5 5.0 4.5 4.0 3.5 3.0 2.5 2.0 1.5 1.0 0.5 0.0
ppm
NH2
NH3OH
11

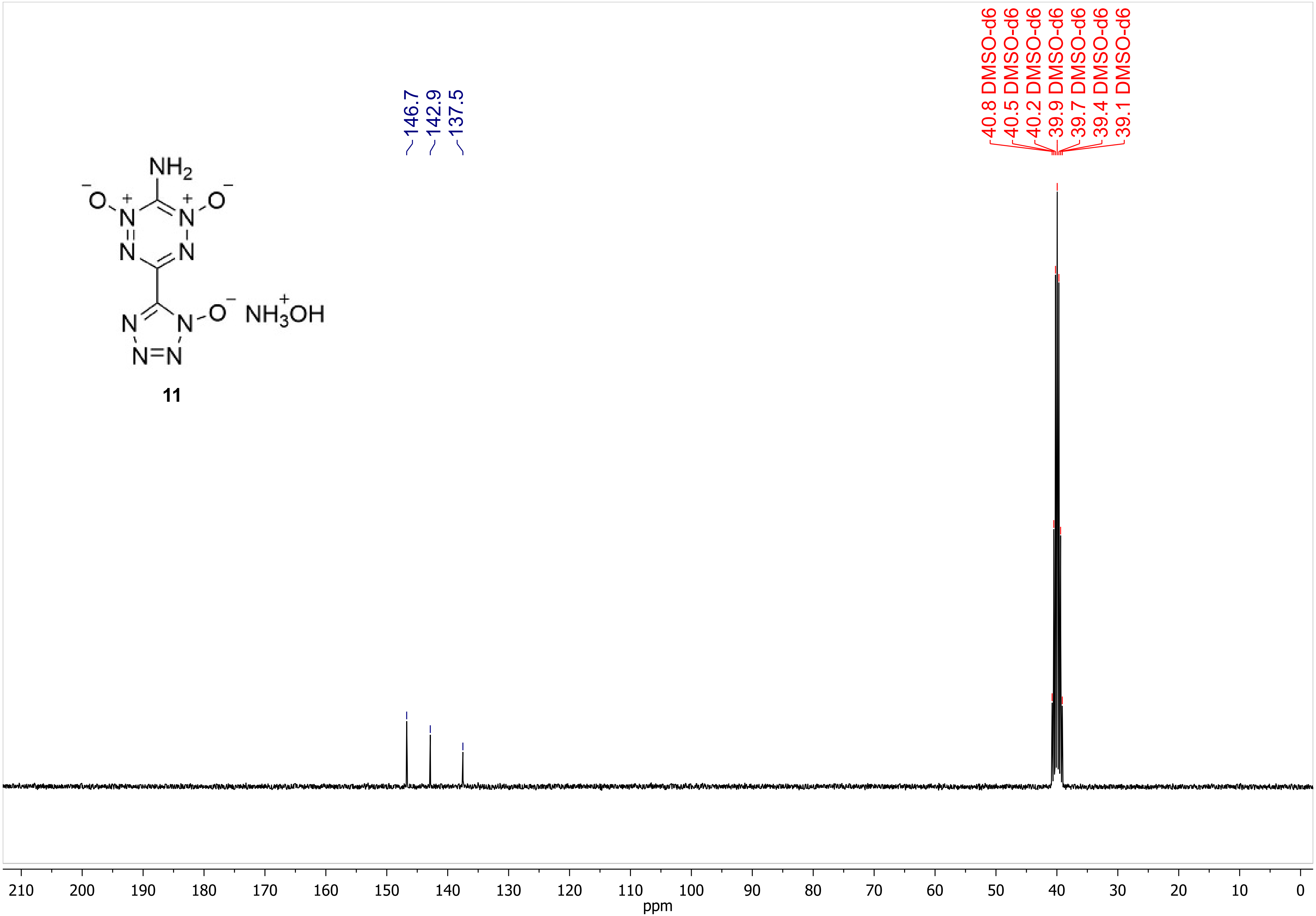

146.7
142.9
137.5
40.8 DMSO-d6
40.5 DMSO-d6
40.2 DMSO-d6
39.9 DMSO-d6
39.7 DMSO-d6
39.4 DMSO-d6
39.1 DMSO-d6
210
200
190
180
170
160
150
140
130
120
110
100
90
80
70
60
50
40
30
20
10
0
ppm

11

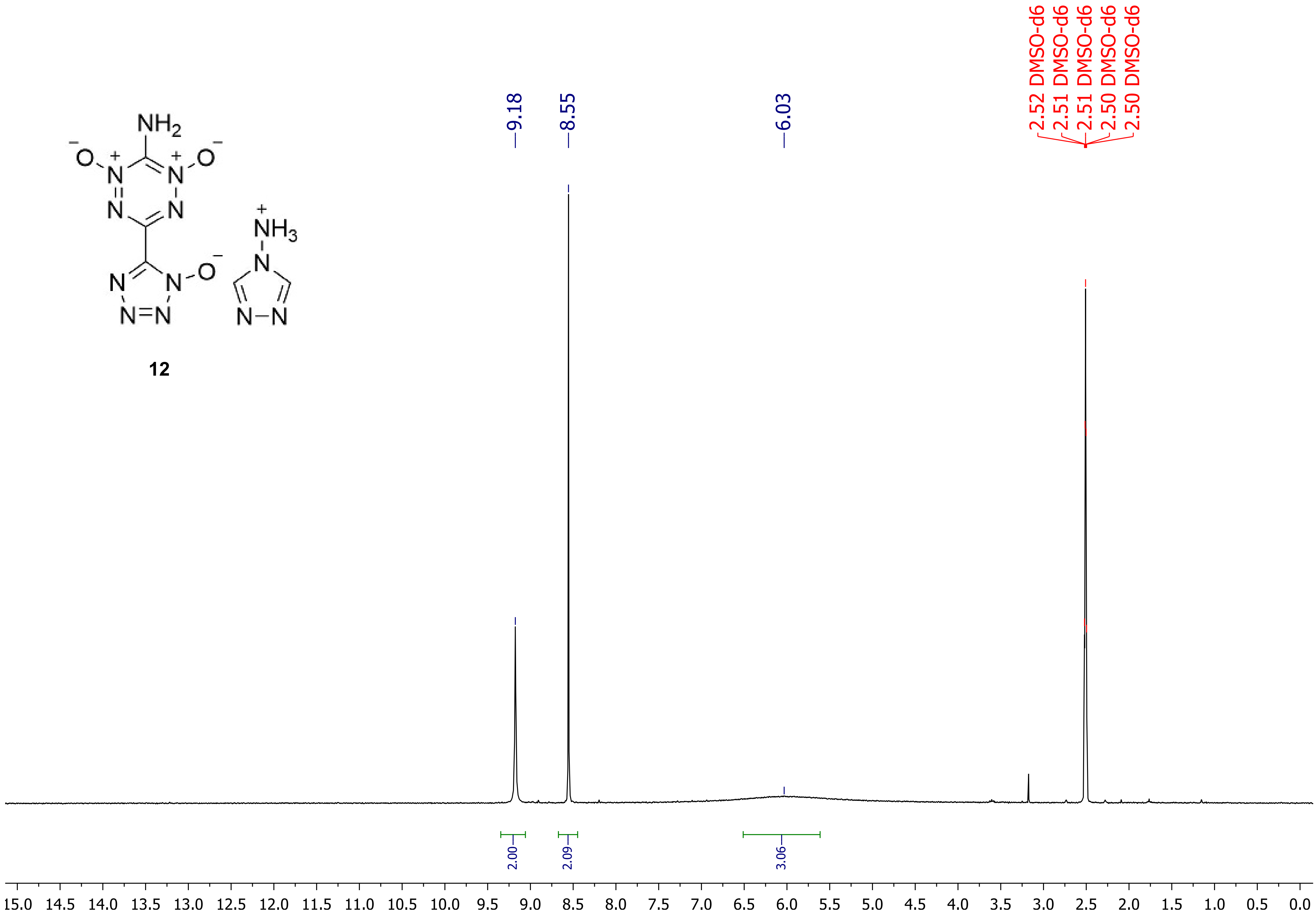

12
9.18
8.55
6.03
2.52 DMSO-d6
2.51 DMSO-d6
2.51 DMSO-d6
2.50 DMSO-d6
2.50 DMSO-d6
2.00
2.09
3.06
15.0 14.5 14.0 13.5 13.0 12.5 12.0 11.5 11.0 10.5 10.0 9.5 9.0 8.5 8.0 7.5 7.0 6.5 6.0 5.5 5.0 4.5 4.0 3.5 3.0 2.5 2.0 1.5 1.0 0.5 0.0

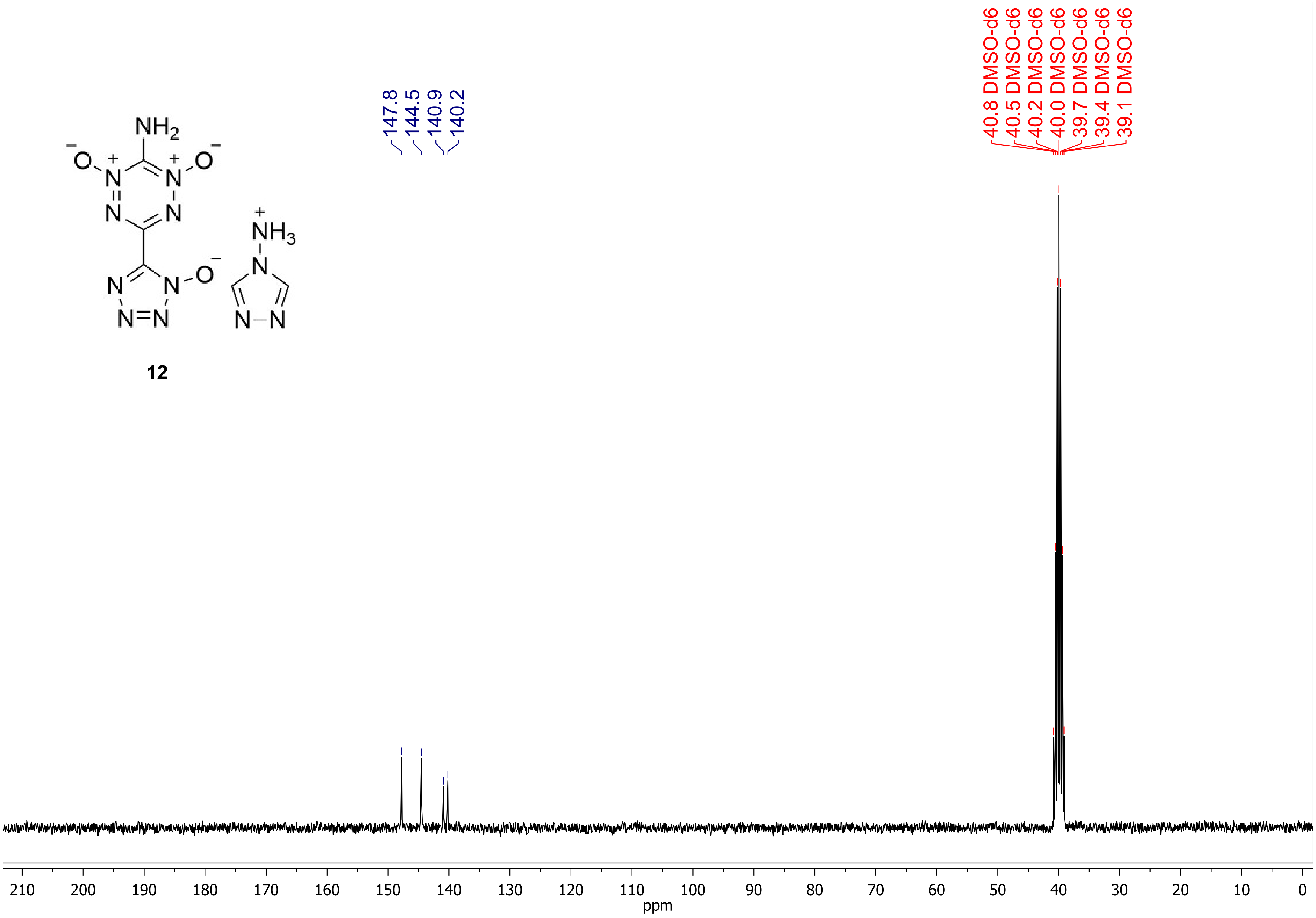

NH2
12
147.8
144.5
140.9
140.2
40.8 DMSO-d6
40.5 DMSO-d6
40.2 DMSO-d6
40.0 DMSO-d6
39.7 DMSO-d6
39.4 DMSO-d6
39.1 DMSO-d6
210 200 190 180 170 160 150 140 130 120 110 100 90 80 70 60 50 40 30 20 10 0
ppm

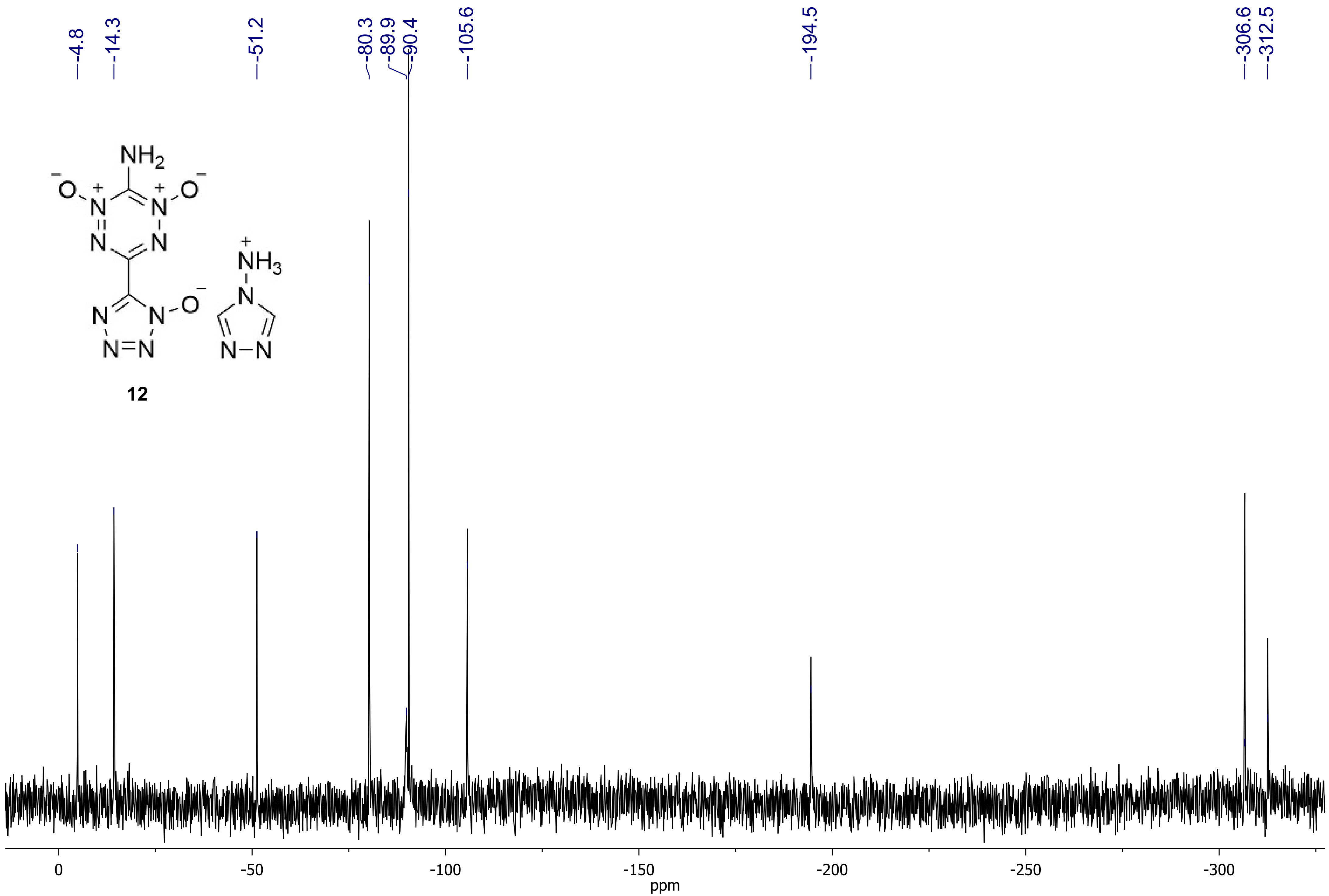

-4.8
-14.3
-51.2
-80.3
-89.9
-90.4
-105.6
-194.5
-306.6
-312.5
NH2
12
NH3
0
-50
-100
-150
-200
-250
-300
ppm
